# Tests of large language models' medical competence and application for clinical decision support of musculoskeletal rehabilitation

**DOI:** 10.3389/fdgth.2025.1719340

**Published:** 2026-02-10

**Authors:** Ruikang Liu, Qiaoling Liu, Qiang Hu, Ruixing Nan, Jian He, Jinru Yang, Jialin Zhang, Guang Yang, Zhaohui Yang, Xiling Xiao, Xiaoxuan Xia, Yongchao Wu

**Affiliations:** Department of Rehabilitation, Union Hospital, Tongji Medical College, Huazhong University of Science and Technology, Wuhan, China

**Keywords:** ChatGPT, DeepSeek, generative artificial intelligence, large language models, rehabilitation

## Abstract

**Objective:**

Large language models (LLMs) are currently abundant and diverse, yet clinicians lack clarity on top performers, with uncertainty about general LLMs' expertise in musculoskeletal rehabilitation. This study aims to investigate the potential and correctness of LLMs in clinical application, and to evaluate whether LLMs could assist primary rehabilitation therapists to prepare for rehabilitation examination.

**Method:**

8 primary doctors and therapists tested 10 LLMs in the first test, 5 senior doctors and therapists assessed answers in the second test, and 5 primary therapists acted as examinees in the third test. We assessed the quality of case analysis based on six different dimensions, including Case Understanding, Clinical Reasoning, Primary Diagnosis, Differential Diagnosis, Treatment Plan Accuracy and Safety, and Guidelines & Consensus.

**Results:**

In the first test, only ERNIE Bot X1 Turbo and Doubao 1.5 pro had accuracy rates of over 90%, and Chinese LLMs had significantly fewer incorrect questions than English LLMs (9.6% vs. 14.8%, *P* < 0.001). In the second test, Doubao 1.5 pro achieved relatively high scores in both cases, and LLMs gained high scores in “Case understanding”, “Clinical Reasoning” and “Diagnosis”. In the third test, primary therapists achieving a mean accuracy rate of 76.9%, and Doubao 1.5 pro improved its accuracy rates to 85.8%.

**Conclusions:**

Doubao 1.5 pro possessed competent ability and application prospects, and was assessed as the best LLM for answering musculoskeletal rehabilitation questions. We also demonstrated that the response quality of local-language LLMs was significantly better than that of English LLMs in answering localized language questions.

## 1 Introduction

Over the past decades, the number of patients requiring musculoskeletal rehabilitation has continuously increased (1). This growth is attributed not only to population expansion and aging but also to the rising prevalence of risk factors for musculoskeletal diseases among young individuals, such as sedentary lifestyles, insufficient physical activity, unhealthy dietary habits, and high body mass index (2–4). However, the development of musculoskeletal rehabilitation encounters numerous challenges: the assessment of dysfunction is primarily based on the patient-reported outcomes from multiple perspectives which often generates high heterogeneity, and the lack of therapist training is also worsening with the increasing aging trend and health demand (5, 6). Meanwhile, rehabilitation therapists must acquire diverse medical knowledge to cope with the diverse diseases and treatments, and ultimately make quick and accurate clinical decisions based on personalized patient needs.

Large language models (LLMs), deep learning models which can understand and generate natural language text, have provided new opportunities to the musculoskeletal rehabilitation development: (1) LLMs can store and integrate massive medical knowledge, and screen quickly before generating an answer; (2) LLMs can deeply analyze medical data of each case to provide individualized diagnostic suggestions and emotional support for different individuals; (3) LLMs have the ability of natural language processing to transform spoken language and unstructured data into standardized medical language and structured data (7–10). Since the advent of the first open-source reasoning model “DeepSeek-R1”, the cost of LLMs has been greatly reduced and the reasoning processes of LLMs have become transparent and interpretable. The realized local deployment of DeepSeek-R1 model in hospital could improve the quality and efficacy of electronic health record writing, disease diagnosis and treatment suggestions, and patient communication (8).

The crucial criterion for measuring the qualification of a rehabilitation therapist is to successfully complete the medical licensing examination. Therefore, the initial assessment of the applicability of LLMs in medical practice should be evaluating their performance in medical tests. As a leading LLM, ChatGPT has demonstrated remarkable potential in achieving passing-level performance on standardized medical tests. Many studies have reported the application prospects of ChatGPT for answering medical questions and clinical reasoning (11–13). However, musculoskeletal rehabilitation is different from other subspecialties. Taking MedQA dataset (from the American medical license examination) and MedMCQA dataset (from the Indian medical entrance examination) as examples, rehabilitation questions are not included in these examinations. Even in the Chinese medical licensing examination, the proportion of rehabilitation questions is less than 1% (14). Therefore, it remains unclear whether LLMs could provide accurate answers to musculoskeletal rehabilitation questions.

Additionally, with the advancement of LLMs' language processing capabilities, many non-English-speaking countries have begun to use LLMs with local languages (15–17). It also remains unclear whether English LLMs can beat local-language LLMs when answering non-English questions. Our study aims to investigate the potential and accuracy of LLM in the clinical applications by comparing the performance of ten popular LLMs in musculoskeletal rehabilitation examination. Meanwhile, we aimed to evaluate whether LLM could assist primary rehabilitation therapists preparing for non-English medical tests.

## 2 Materials and methods

**Figure 1:**
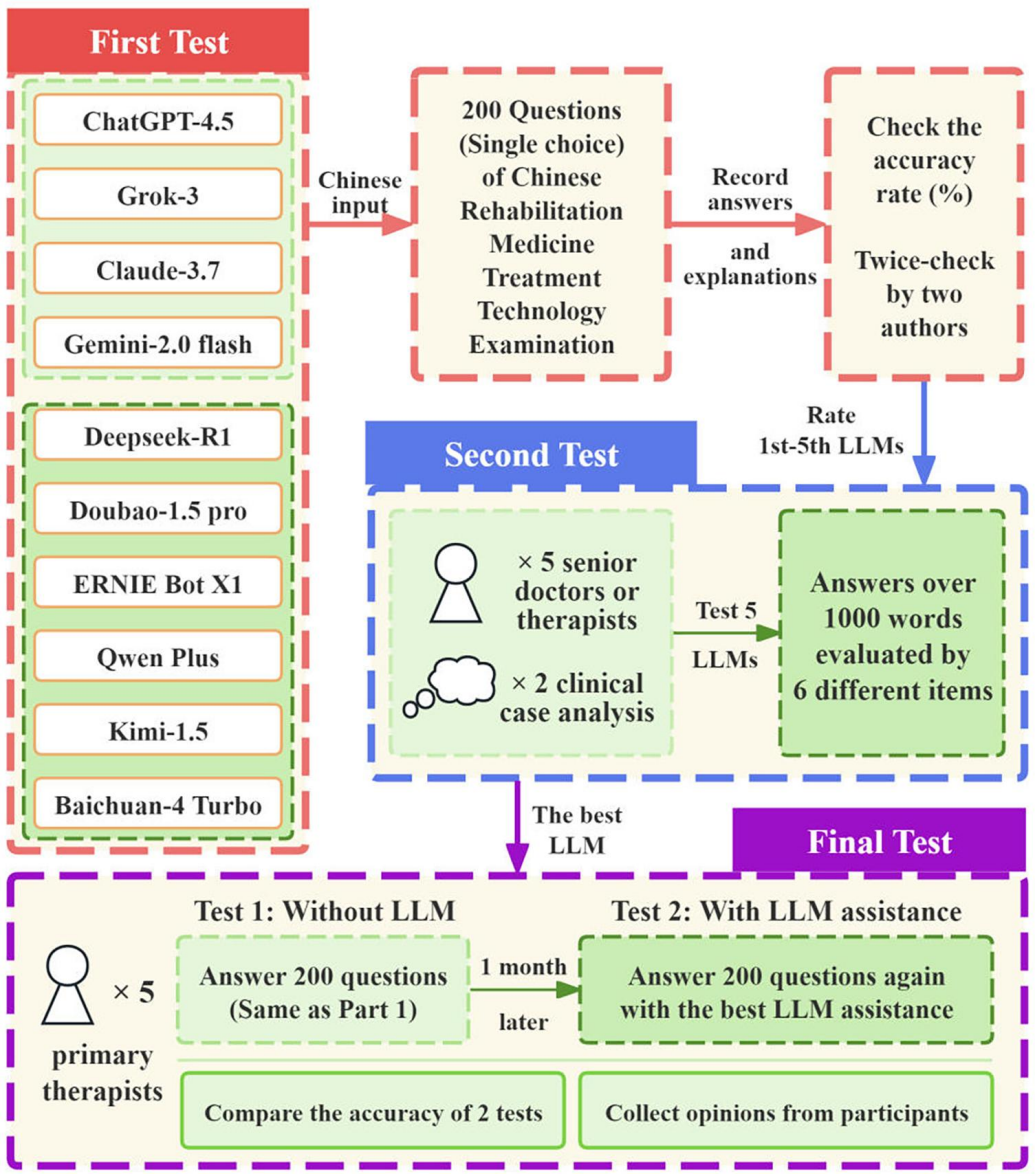
The flowchart of the overall study design.

Our study did not involve the collection of patient data or interventions on patients. Therefore, the ethical approval was waived by the Ethics Committee of Huazhong University of Science and Technology Tongji Medical College Affiliated Union Hospital. All procedures were conducted in accordance with the Declaration of Helsinki. This study did not involve real patient data, therefore no informed consent form is required. Figure 1 illustrates the flowchart of the overall study design.

### 2.1 First test

Ten popular LLMs were tested in this study, including: (1) ChatGPT-4.1 (Open AI), (2) Grok 3 (xAI), (3) Claude 3.7 Sonnet (Anthropic), (4) Gemini 2.5 Flash thinking (Google), (5) DeepSeek-R1 (DeepSeek), (6) Doubao 1.5 pro (ByteDance), (7) ERNIE Bot X1 Turbo (Baidu), (8) Qwen 3.0 (Alibaba), (9) Kimi 1.5 (Moonshot AI), and (10) Baichuan 4 Turbo (Baichuan AI). Detailed information on all the included LLMs is provided in Table 1.

**Table 1:** Detailed information on the included LLMs.

LLMs	Language	Institutes	Release date	Accessed website
GPT 4.1	English	Open AI	May 2025	https://chatgpt.com/
Grok 3	English	xAI	Feb 2025	https://grok.com/
Claude 3.7 Sonnet	English	Anthropic	Feb 2025	https://claude.ai/new
Gemini 2.5 flash	English	Google	Jun 2025	https://gemini.google.com/app
DeepSeek-R1	Chinese	DeepSeek	May 2025	https://chat.DeepSeek.com/
Doubao 1.5 pro	Chinese	ByteDance	Mar 2025	https://console.volcengine.com/
ERNIE Bot X1 Turbo	Chinese	Baidu	Apr 2025	https://yiyan.baidu.com/
Qwen 3.0	Chinese	Alibaba	Apr 2025	https://www.aliyun.com/product/tongyi
Kimi 1.5	Chinese	Moonshot AI	Jan 2025	https://kimi.moonshot.cn/
Baichuan 4 Turbo	Chinese	Baichuan AI	Oct 2024	https://platform.baichuan-ai.com/playground

ChatGPT-4.1, Grok 3, Claude 3.7 Sonnet, and Gemini 2.5 Flash Thinking emerge as the latest iterations of internationally renowned general LLMs. The remaining six models are Chinese LLMs: DeepSeek-R1, as the first open-source reasoning model, has been locally deployed in hundreds of hospitals; Doubao 1.5 pro, ERNIE Bot X1 Turbo, and Qwen 3.0 are LLMs developed by major Chinese internet corporations, which were trained with extensive datasets and exhibit robust response capabilities; Kimi 1.5 is another popular Chinese model, excelling in long-context retention; Baichuan 4 Turbo stands out as a medical-focused LLM, trained on 1 trillion tokens of clinical records, textbooks, and guidelines, enabling perfect diagnostic reasoning within healthcare verticals.

The authoritative musculoskeletal rehabilitation-related questions for LLM testing were sourced from the real exams of *Chinese Rehabilitation Medicine Treatment Technology Examination*, which is a professional certification test designed for primary rehabilitation therapists. A passing threshold was defined as ≥60% accuracy, and lower scores were deemed failing. We selected 200 distinct musculoskeletal rehabilitation-related single-choice questions (from exams held between 2011 and 2024) to evaluate the performance of the 10 selected LLMs. These questions cover the areas of foundational theoretical knowledge (e.g., muscle physiology, biomechanics of joint movement), clinical decision (e.g., selecting appropriate therapeutic exercises for post-fracture rehabilitation), and complex case analysis (e.g., designing multi-modal treatment plans).

Each LLM was evaluated using a newly created account without any pre-existing interaction history, ensuring that no prior dialogue data would influence the answers. The musculoskeletal rehabilitation questions and options were input into all 10 selected LLMs in Chinese. For each question, the models were instructed to provide both answers and detailed explanation. Answers and explanations for each question from every chatbot were cross-referenced with official answers to determine the accuracy rate. All questions were independently tested by two authors, and questions that gave different answers when queried multiple times were defined as wavering questions.

### 2.2 Second test

The second test aimed to assess the clinical reasoning depth of the top five LLMs from the first test through complex case analysis. Two authentic clinical case scenarios in musculoskeletal rehabilitation (Case 1 lumbar disc herniation, Case 2 cervical spondylotic radiculopathy) were selected by five senior doctors and therapists with over 10 years of experience. Both cases included age, gender, chief complaint, current medical history, past medical history, and physical examination (see Supplementary File S1). To unify and standardize the answer, each model was required to respond the primary diagnosis, differential diagnoses, additional diagnostic tests and rehabilitation treatment plan over 1,000 words. Then, five senior doctors and therapists would evaluate and score these answers based on six different items (see Supplementary File S2), which presents scoring criteria for clinical case assessment across multiple dimensions, including Case Understanding, Clinical Reasoning, Primary Diagnosis, Differential Diagnosis, Accuracy and Safety of treatment plan, and Guidelines & Consensus.

### 2.3 Final test

We further evaluated the performance of five primary rehabilitation therapists (with clinical experience ≤3 years) using the same 200 musculoskeletal rehabilitation questions. We required them to undergo two rounds of testing and recorded their accuracy rates, one round required independent completion, and the other allowed assistance from the LLM which performed the best in previous comparison. After a 1-month interval to minimize carryover effects, participants were required to take the second round of testing. All participants were informed that LLM's answers were not guaranteed to be correct, and they were required to independently verify the model's responses and explanations using their professional judgment.

### 2.4 Statistical analysis

The accuracy rate (%) for each chatbot and participant was calculated as the proportion of correct answers to the total number of questions. Chi-square tests were utilized to compare the accuracy rate between different LLMs. Paired t-tests were applied to compare the accuracy rate of each participant without and with LLM's assistance. SPSS 26.0 was used for these statistical analyses. *P* < 0.05 was considered to be statistically significant.

## 3 Result

### 3.1 First test

**Figure 2:**
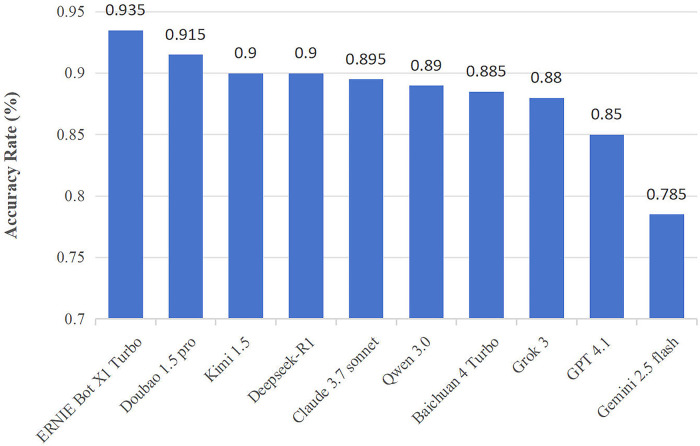
Accuracy of LLMs in the first test. ERNIE Bot X1 Turbo and Doubao 1.5 pro had accuracy rates of over 90%, and the average accuracy of Chinese models was significantly higher than English models.

The performance of ten LLM-chatbots with Chinese input in the *Chinese Rehabilitation Medicine Treatment Technology Examination* is depicted in Figure 2. The accuracy rate of all LLMs was higher than the pass mark, and two models had accuracy rates of over 90% (ERNIE Bot X1 Turbo: 93.5%; Doubao 1.5 pro: 91.5%). Gemini 2.5 Flash performed the worst (78.5%), followed by ChatGPT-4.1 (85.0%), both of their accuracy rates were significantly lower than ERNIE Bot X1 Turbo (P_Gemini_ < 0.001, P_ChatGPT_ = 0.015). Besides, the average accuracy of Chinese models was significantly higher than that of English models (90.4% vs. 84.3%, *P* < 0.001).

We further analyzed the number and types of incorrect questions, and we present detailed data in Table 2. Higher number of wavering questions reflects the instability of model answers, Doubao 1.5 pro impressively has no wavering questions, while ERNIE Bot X1 Turbo has five. In comparison, Gemini 2.5 Flash and Qwen 3.0 have more than 10 wavering questions. As for the question type, English LLMs had a total of 118 (14.8%) incorrect questions (73 related to theoretical knowledge and 45 for clinical decision/case analysis), while Chinese LLMs had a total of 115 (9.6%) incorrect questions (77 related to theoretical knowledge and 38 for clinical decision/case analysis) with both significant differences (P_total_ < 0.001, P_theoretical knowledge_ = 0.022, P_clinical decision/case analysis_ = 0.004). As for the disease type, English LLMs had a total of 36 (12.9%) incorrect questions related to spine and 27 (13.5%) for lower limb, while Chinese LLMs had 29 (6.9%) and 24 (8.0%) respectively, with also both significant differences (P_spine_ = 0.008, P_lower limb_ = 0.047).

**Table 2:** Primary outcomes of the first test—performance comparison between English and Chinese LLMs.

Models	Error questions	Wavering questions	Question type		Disease type			
			Theoretical knowledge	Clinical decision/Case analysis	Upper limb	Spine	Lower limb	Others
			(*N* = 140)	(*N* = 60)	(*N* = 50)	(*N* = 70)	(*N* = 50)	(*N* = 30)
English LLMs								
GPT 4.1	30	4	19	11	9	7	7	7
Grok 3	24	4	14	10	8	7	4	5
Claude 3.7 Sonnet^#^	21	3	15	6	4	7	7	3
Gemini 2.5 flash	43	12	25	18	12	15	9	7
Total *N* (%)	118 (14.8)	23 (2.9)	73 (13.0)	45 (18.8)	33 (16.5)	36 (12.9)	27 (13.5)	22 (18.3)
Chinese LLMs								
DeepSeek-R1^#^	20	2	13	7	6	4	5	5
Doubao 1.5 pro^#^	17	0	10	7	7	3	4	3
ERNIE Bot X1 Turbo^#^	13	5	9	4	2	4	4	3
Qwen 3.0	22	10	16	6	7	4	4	7
Kimi 1.5^#^	20	4	13	7	5	6	1	8
Baichuan 4 Turbo	23	5	16	7	6	8	6	3
Total N (%)	115 (9.6)	26 (2.2)	77 (9.2)	38 (10.6)	33 (11.0)	29 (6.9)	24 (8.0)	29 (16.1)
*P* value**	**<0.001** *	0.315	**0.022** *	**0.004** *	0.075	**0.008**	**0.047** *	0.616

* *P* value ≤ 0.05.

** *P* value (English LLMs vs. Chinese LLMs).

# The rate 1st-5th models in primary evaluation.

Bold means *p value ≤ 0.05.

### 3.2 Second test

**Figure 3:**
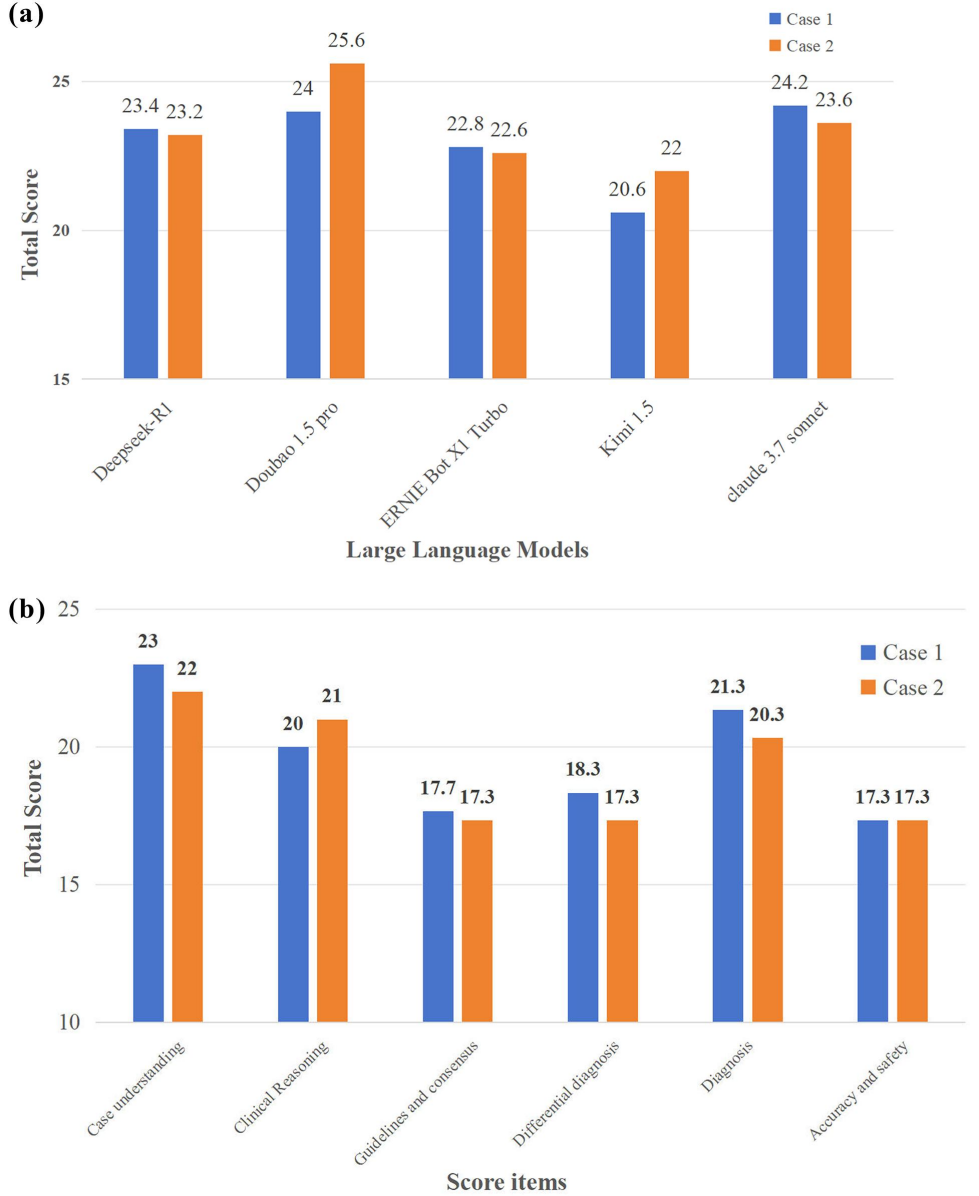
**(a)** Overall performance of LLMs in the second test. **(b)** Different performance across six items. Doubao 1.5 pro achieved relatively high scores in both cases, and LLMs showed the capabilities of “Case understanding”, “Clinical reasoning” and “Diagnosis”.

The second test probed the top-five LLMs (DeepSeek-R1, Doubao 1.5 pro, ERNIE Bot X1 Turbo, Kimi 1.5, Claude 3.7 Sonnet) from the first test for clinical reasoning depth via complex musculoskeletal rehabilitation cases (lumbar disc herniation, cervical spondylotic radiculopathy). Senior doctors/therapists scored responses on six items following the criteria in Supplementary File S2. Figure 3a demonstrates the overall performance of these LLMs across cases: Doubao 1.5 Pro achieved relatively high scores in both cases, especially in Case 2 (25.6 points), while Claude 3.7 Sonnet also performed well in Case 1 (24.2 points). DeepSeek-R1's scores were relatively close but lower than the top two models. Figure 3b illustrates performance across six items. Notably, “Case understanding” yields the highest scores (Case 1 23 points and Case 2 22 points), reflecting the strength of LLMs in grasping case details and interpreting information. Meanwhile, “Clinical Reasoning” and “Diagnosis” also achieve high scores, indicating the capability in logical clinical deduction and formulating diagnostic conclusions. However, “Guidelines and consensus” and “Accuracy and safety” obtain relatively low scores. This suggests two key issues: LLMs may struggle to consistently align with published medical guidelines and clinical consensus, and the treatment plans suggested by LLMs are not reliable enough for direct clinical guidance.

### 3.3 Final test

Given the results of the first and second tests, the Doubao 1.5 pro model demonstrated the best comprehensive performance, so it was selected for the final test. Table 3 illustrates therapists' performance changes with LLM assistance. All five primary therapists completed the identical test in the first-round assessment, achieving a mean accuracy rate of 76.9% (ranging from 71% to 82%). Across participants, accuracy improved from Test 1 to Test 2, with an average increase of 8.9% (rising from 76.9% to 85.8%). Regarding answer adjustments, “Correct” changes averaged 20.8, while “Wrong” changes were notably lower, with a mean of 3. These “Wrong” adjustments occurred when the LLM provided wrong answers, and participants blindly accepted them, consequently reversing their previously correct answers. Overall, LLM assistance effectively enhanced accuracy, but the risk of participants adopting LLM-generated wrong answers without sufficient verification remains a critical concern.

**Table 3:** Therapists' improvements and answer changes with LLM assistance.

Participant	Accuracy (%)			Change question*	
	Test 1	Test 2	Improvement	Correct	Wrong
Average	76.9	85.8	8.9	20.8	3
Therapist #1	80.5	88	7.5	20	5
Therapist #2	71	83	12	28	4
Therapist #3	82	93	11	23	1
Therapist #4	77.5	85	7.5	19	4
Therapist #5	73.5	80	6.5	14	1

* Correct means change wrong answers to correct ones, and Wrong means change correct answers to wrong ones.

## 4 Discussion

Based on different question types and disease categories of the *Chinese Rehabilitation Medicine Treatment Technology Examinatio*n and clinical cases, we design three tests to comprehensively evaluate the medical competence, logical reasoning, and ability for primary therapist training of LLM. In general, we conclude that Doubao 1.5 pro is the LLM with the best ability and application prospects for answering musculoskeletal rehabilitation questions, and helps primary therapists improve accuracy currently. Besides, we demonstrate the response quality of Chinese LLM is significantly better than that of English LLM in answering Chinese questions. However, we found that current LLM responses still contain errors and may mislead users to change originally correct answers. While we highlighted this risk, our small sample (five junior therapists) limited robust quantification. Root causes include therapists' overtrust—stemming from poor awareness of model limitations and inadequate verification—exacerbated by LLMs' confident tone. Model-side errors likely arise from outdated rehabilitation guidelines in training data and poor handling of ambiguous terms (e.g., subtle diagnostic criteria differences). Mitigation strategies include mandating LLMs to cite guidelines/evidence and requiring human review for high-risk decisions (e.g., disease grading, treatment dosage). These measures boost LLM clinical safety, aligning with our goal of bridging model capability and clinical practice. Therefore, it indicates that general LLMs still need to supplement professional knowledge of musculoskeletal rehabilitation to build a more comprehensive knowledge base, so as to avoid misleading users in real-world clinical diagnosis and treatment.

Notably, the accuracy of LLMs in clinical decision and case analysis questions is significantly lower than that in theoretical knowledge questions. This suggests that although LLMs demonstrate stable knowledge memorization and retrieval capabilities, they exhibit notable limitations in reasoning about complex clinical scenarios. Overall, four common types of high-risk incorrect questions can be summarized. Firstly, grading-related questions involving disease severity or diagnostic-stage judgments, where LLMs often misclassify due to imprecise understanding of grading criteria. Secondly, One-character discrepancy questions with minor lexical variations in key terms (such as disease names or clinical terminology), which frequently lead to misjudgment. Thirdly, questions with subtle differences in options where options vary only in trivial details could be hardly distinguished. Lastly, specific treatment dosage questions regarding parameters like instrument intensity settings or rehabilitation plan on individual patients. These findings highlight the need to enhance the depth and precision of clinical knowledge in LLMs, providing clear directions for future model optimization and clinical application.

Prior researches have extensively evaluated the response accuracy of GPT 3.5 and GPT 4.0 in various specialized medical examinations, and conducted comparisons against other English LLMs, and most of studies regarded GPT 4.0 as the strongest LLM in medical examination (18–22). However, the second and final tests of our study are designed to simulate the real clinical scenarios, while our institution's doctors and therapists only use Chinese during work and test, so we only assessed the localized (Chinese) answer ability of LLMs. Nevertheless, the methodology and conclusion of our study could still offer insights for further localized optimization and application of LLM researches.

Furthermore, several researches recently demonstrated that Chinese LLMs may have similar ability with English LLMs in answering Chinese medical examinations: Yuan et al. focused on liver cancer-related clinical guideline questions, and demonstrated ERNIE Bot 4.0 achieved an accuracy of 89.47%, while GPT 4.0 reached 92.11%, showing comparable performance between Chinese and English models in certain medical knowledge domains (16). In nursing-related examinations, Zhu et al. reported that Qwen 2.5 obtained a 90.8% accuracy rate on 1,200 choice questions from the 2019–2023 Chinese National Nursing Licensing Examination, beating GPT-4o in nursing knowledge assessment (23). Zhou et al. also found that DeepSeek-R1 and GPT-4o gained similar scores in spine surgery-related tests (DeepSeek-R1: 7.2–9.0, GPT-4o: 8.2–9.5) (24). Moreover, in a surgery-related test with 806 choice questions from Medical Vision World, ERNIE Bot 4.0 achieved an accuracy range of 78% which beating GPT 4.0 (15). In conclusion, these findings also collectively illustrate that Chinese LLMs are capable of matching or even surpassing the performance of English LLMs when answering questions with local-language.

Through testing five junior rehabilitation therapists, we have convincingly demonstrated that LLMs can assist in enhancing exam performance. The therapists achieved an average accuracy rate of 76.9% when answering questions independently, and it increased to 85.8% with the assistance of the Doubao 1.5 pro model. These outcomes indicate that LLMs can provide effective references for examinees and broaden their thinking. Despite the risk of blindly accepting incorrect answers from LLMs, its positive role in knowledge supplementation and problem-solving inspiration cannot be denied, offering a new pathway for improving exam capabilities of primary therapists.

Our study evaluated the accuracy of ten popular LLMs in answering musculoskeletal rehabilitation questions from the *Chinese Rehabilitation Medicine Treatment Technology Examination*, as well as their knowledge and reasoning capabilities in real-world clinical reasoning. Overall, most LLMs demonstrated broad knowledge bases and reasoning abilities in addressing musculoskeletal rehabilitation questions. Notably, the Doubao 1.5 pro model showed substantial potential in providing knowledge support for primary therapists and improving exam accuracy, which could potentially revolutionize medical training and help alleviate the shortage of rehabilitation therapists. However, while LLMs can currently process medical information effectively and provide appropriate answers to questions, they cannot substitute critical thinking, innovation, and creativity, which experienced physicians must possess. To develop specialized rehabilitation medical LLMs in the future, we must address challenges such as the scarcity of specialized data and the complexity of annotation. Given our findings that LLMs excel in theoretical knowledge retrieval but face limitations in complex clinical reasoning and high-risk decision-making (e.g., disease grading, treatment dosage adjustment), defining clear boundaries for LLM application is imperative. Specifically, tasks requiring subjective judgment, individualized plan formulation, or critical clinical decisions should mandate mandatory human oversight to mitigate risks of incorrect AI outputs. In contrast, LLM can be entrusted with auxiliary tasks such as medical knowledge query, case literature retrieval, and preliminary answer suggestions to enhance work efficiency. Establishing such a collaborative framework not only addresses the current risk of overtrust in AI but also maximizes the complementary advantages of human clinical experience and AI's information processing capabilities. Additionally, an iterative optimization mechanism involving specialized physicians, ethicists, and data scientists should be established. Only through such measures can we propel LLMs to make a substantial leap from “medical knowledge Q&A” to “specialized decision support”.

## 5 Study limitations

This study has certain limitations. Firstly, the 10 LLMs were selected primarily based on their popularity and ease of use; some unpopular models were not included, which may introduce selection bias. Secondly, the “illusion” of artificial intelligence cannot be ignored. When the model fabricates facts or gives different responses upon multiple queries, it may mislead users without professional backgrounds. Thirdly, current models generally lack citations to clinical guidelines in their responses. If model optimization can be combined with a dynamic guideline update mechanism, it is expected to achieve a balance between standardized and personalized diagnosis and treatment. Fourthly, we did not stratify analysis by question difficulty—due to no standardized classification in the question bank and inconsistent team opinions on objective evaluation—limiting precise identification of LLMs' strengths across difficulty tiers.

## 6 Conclusion

Doubao 1.5 pro was the LLM with the best ability and application prospects for answering musculoskeletal rehabilitation questions, and helped primary therapists improve accuracy. We also demonstrated the response quality of local-language LLMs was significantly better than that of English LLMs in answering localized questions.

## Data Availability

The raw data supporting the conclusions of this article will be made available by the authors, without undue reservation.
