# Supplementary material for: Tests of large language models' medical competence and application for clinical decision support of musculoskeletal rehabilitation
Source: Front Digit Health. 2026 Feb 10;7:1719340. doi: 10.3389/fdgth.2025.1719340 (PMC12929487; doi:10.3389/fdgth.2025.1719340)
Supplement: Supplementary file 1 [file Table1.docx]

| Scoring items | Scoring criteria | | |
| --- | --- | --- | --- |
|  | 1-2 point | 3-4 point | 5 point |
| Case understanding | Fail to identify core elements, severely misinterpret key information, and provide irrelevant explanations | Grasp main structure and key information, with minor inaccuracies in details | Fully and accurately understand all content, including implicit clues, matching experts |
| Clinical Reasoning | Reasoning violates clinical logic, leads to irrelevant conclusions and goes against medical knowledge | Follow basic diagnosis process, reasonable analysis but incomplete differential diagnosis | Rigorous logic, consider rare manifestations and individual differences, expert-level conclusions |
| Diagnosis | Diagnoses are absurd and contradictory to cases, clinically unacceptable | Diagnoses based on main information with uncertainties or ambiguity, divisive acceptance | Comprehensive and accurate diagnoses with staging/severity, detailed enough for clinical guidance |
| Differential diagnosis | List irrelevant diagnoses without evidence | Identify multiple relevant causes with brief analysis | Prioritize by probability/severity, include rare diseases |
| Accuracy and safety of treatment plan | Completely inconsistent with the patient's condition, uses contraindicated treatments that may cause severe adverse reactions, or lacks basis, has safety hazards and ignores individual differences | Basically conform to routine principles without obvious contraindications but lack specificity, and formulate a personalized plan | Precisely formulate rehabilitation plans combining disease characteristics, individual differences and the latest research, arrange treatment scientifically |
| Guidelines and consensus | Completely contradict guidelines, with critical errors that may mislead practice | Follow main framework but lack details or updated content, adhere to guidelines strictly | Precisely apply guidelines, combine individual needs, and handle complex cases reasonably |
