# Supplementary material for: Tests of large language models' medical competence and application for clinical decision support of musculoskeletal rehabilitation
Source: Front Digit Health. 2026 Feb 10;7:1719340. doi: 10.3389/fdgth.2025.1719340 (PMC12929487; doi:10.3389/fdgth.2025.1719340)
Supplement: Supplementary file 2 [file Datasheet1.docx]

**Case 1**

Prompt: You are an experienced clinical physician in the department of rehabilitation. Facing the following patient's condition, please answer the four questions in a total of 800-1000 Chinese characters: (1) What is the patient's preliminary diagnosis? (2) List 4 main differential diagnoses. (3) Write the auxiliary examinations for a clear diagnosis. (4) Briefly describe the rehabilitation treatment methods and principles for preventing recurrence.

Patient Information: Zhang XX, female, 48 years old. Repeated left lumbocrural pain for 2 years, aggravated with left lower limb numbness for 1 week.

Current medical history: Two years ago, she developed left lumbar soreness after carrying heavy objects, gradually radiating to the left buttock and posterior thigh, which relieved after bed rest. Since then, the symptoms have recurred, worsening after fatigue or prolonged sitting, occasionally accompanied by numbness in the lateral left calf. One week ago, when bending over to pick up an object, she suddenly experienced severe left lumbocrural pain like an "electric shock," radiating to the anterolateral left calf and medial dorsum of the foot. The pain intensified during coughing and sneezing and alleviated in the supine flexed knee position. No urinary or fecal incontinence or saddle area numbness was noted.

Past Medical History: Healthy, no infectious diseases, no trauma or surgical history.

Occupation: Driver.

Physical Examination:(1) Painful facial expression, passive posture, antalgic gait, left lumbar scoliosis compensation, tension in the left sacrospinal muscles. (2) Tenderness at the interspinous space of L4/L5 and left paravertebral area (+), with pain radiating to the left buttock, posterior thigh, anterolateral calf, and medial dorsum of the foot when pressed. Skin temperature is normal, no redness or swelling. (3)No obvious tenderness in the lower limbs. Lumbar flexion and left lateral flexion are limited. Positive Pick-up Test. (4) Left straight leg raising test positive at 30°, with positive strengthening test. (5) Decreased pain and touch sensation in the anterolateral left calf and medial dorsum of the foot. (6) Left great toe dorsiflexion muscle strength grade 3, tibialis anterior muscle strength grade 4, remaining muscle strengths normal. (6) Normal left patellar reflex, weakened Achilles reflex. (7) ADL: Barthel Index 80 points; Oswestry Disability Index Questionnaire 45 points.

**Case 2**

You are an experienced clinical physician in the department of rehabilitation. Facing the following patient's condition, please answer the four questions in a total of 800-1000 Chinese characters: (1) What is the patient's preliminary diagnosis? (2) List 4 main differential diagnoses. (3) Write the auxiliary examinations for a clear diagnosis. (4) Briefly describe the rehabilitation treatment methods and principles for preventing recurrence.

Patient Information: Li XX, male, 55 years old. Recurrent cervicobrachial soreness for 3 years, exacerbated with radiating numbness and pain in the right upper limb for 2 weeks.

Current medical history: Three years ago, he developed neck and shoulder stiffness and pain without obvious cause, which relieved with activity. Symptoms have recurred frequently in the past six months, worsening with fatigue or cold exposure, occasionally accompanied by numbness in the right thumb and radial side of the forearm. Two weeks ago, after sedentary desk work, he suddenly experienced severe right cervicobrachial pain radiating along the lateral right upper arm, radial forearm to the thumb, described as "burning-like." Pain intensified with coughing or head rotation and partially relieved with rest. No dizziness, headache, cotton-stepping sensation, or thoracic/abdominal band-like sensation.

Past Medical History: 5-year history of hypertension, stably controlled with medication.

Occupation: Programmer.

Physical Examination: (1) Limited neck mobility; tenderness at right C5-6 paravertebral region (+), radiating to the right shoulder and upper limb. (2) Positive right brachial plexus traction test; positive right Spurling test. (3) Decreased pain sensation over the radial forearm and dorsal thumb; diminished biceps reflex; right abductor pollicis brevis muscle strength: Grade 4; remaining muscle strengths normal. (4) Bilateral Hoffmann sign (-); Babinski sign (-). (5) ADL: Barthel Index 90 points; Neck Disability Index (NDI) Questionnaire: 28 points.
